# Augmenting Function for Infarction from Infection: Impella 2.5 for Ischemic Cardiogenic Shock Complicating Sepsis

**DOI:** 10.1155/2017/8407530

**Published:** 2017-02-05

**Authors:** Praveen George, Mukta C. Srivastava, Jonathan Ludmir, Robert M. Reed, Semhar Z. Tewelde, Anuj Gupta, Michael T. McCurdy

**Affiliations:** ^1^Department of Internal Medicine, University of Maryland School of Medicine, Baltimore, MD, USA; ^2^Division of Cardiology, University of Maryland School of Medicine, Baltimore, MD, USA; ^3^Division of Pulmonary and Critical Care Medicine, University of Maryland School of Medicine, Baltimore, MD, USA; ^4^Department of Emergency Medicine, University of Maryland School of Medicine, Baltimore, MD, USA

## Abstract

Cardiac dysfunction is a common complication of sepsis in individuals with preexisting coronary disease and portends a poor prognosis when progressing to ischemic cardiogenic shock. In this setting, maximal medical therapy in isolation is often inadequate to maintain cardiac output for patients who are poor candidates for immediate revascularization. Furthermore, the use of vasopressors and inotropes increases myocardial demand and may lead to further injury. Percutaneous ventricular assist devices provide a viable option for management of severe shock with multiorgan failure. The Impella is one of several novel mechanical support systems that can effectively augment cardiac output while reducing myocardial demand and serve as a bridge to recovery from severe hemodynamic compromise. This case report describes the successful utilization of the Impella 2.5 in a patient with baseline profound anemia and coronary artery disease (CAD) presenting in combined distributive and cardiogenic shock associated with a type 2 myocardial infarction complicating sepsis.

## 1. Introduction

The Impella is a temporary mechanical support device that can bridge a patient with severe cardiac dysfunction to either recovery or further therapy. Conventionally, these devices are utilized in patients undergoing high-risk percutaneous coronary intervention [1, 2] or with acute cardiogenic shock [3]. We describe the successful utilization of the Impella 2.5 in a patient with baseline profound anemia and coronary artery disease presenting in combined distributive and cardiogenic shock complicating sepsis.

## 2. Case Presentation

A 66-year-old woman presented to an outside hospital with several weeks of progressive shortness of breath and weakness. Vitals on arrival were heart rate of 108 beats per minute, blood pressure of 97/59 mmHg, and respiratory rate of 30 breaths per minute without hypoxemia. Physical exam revealed respiratory distress, no abdominal tenderness, diminished pulses throughout, and no evidence of bleeding. Notable laboratory data were 19,000 white blood cells/*μ*L, hemoglobin 4.4 g/dL, troponin 4 ng/mL, lactate 10.6 mmol/L, and urinalysis consistent with urinary tract infection. Electrocardiogram revealed inferolateral ST depressions, and a chest radiograph demonstrated bilateral pulmonary infiltrates. She received broad-spectrum antibiotics and four units of packed red blood cells but required intubation for acute hypoxemic respiratory failure, progressive confusion, and shock refractory to stress-dose steroids and vasopressors. The patient was transferred to our medical intensive care unit for further care.

**Figure 1:**
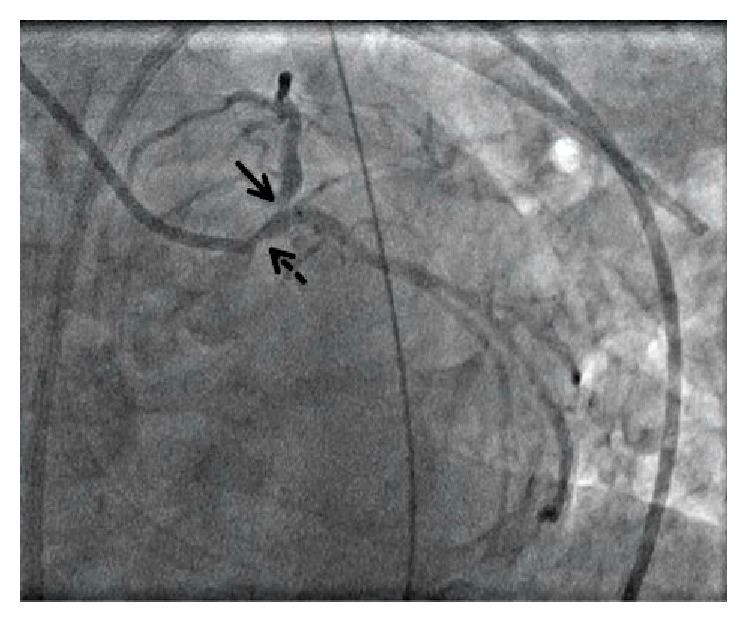
Left heart catheterization demonstrating left main disease (dashed) and 70% LAD ostial lesion (solid).

**Figure 2:**
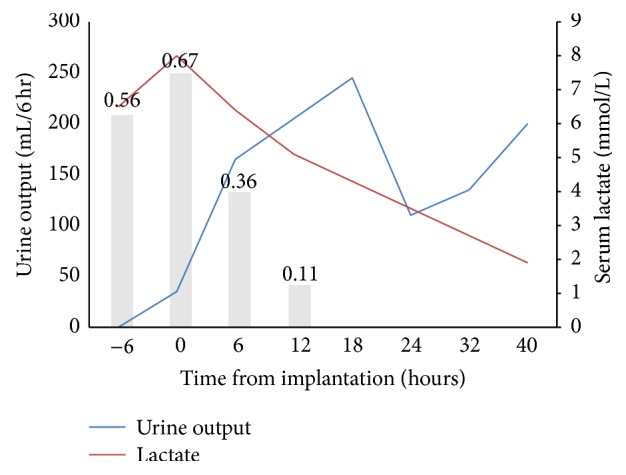
Graphic trend of urine output (blue line), serum lactate (red line), and total vasopressor requirement (gray bars) at six-hour intervals from Impella deployment. ^*∗*^Total vasopressor requirement expressed in mcg/kg/min, as calculated by norepinephrine dose equivalence [4].

On arrival to our intensive care unit, the patient presented intubated and on multiple pressors but had preserved mentation and purposeful movement. Despite medical optimization with the addition of an inotrope and titration of vasopressors to a mean arterial pressure of ≥65 mmHg, she developed anuric renal failure. Repeat blood work showed persistently elevated lactate of 8.0 mmol/L, worsening leukocytosis to 56,000 cells/*μ*L, and troponin markedly elevated at 105 ng/mL. Chest radiograph demonstrated diffuse bilateral infiltrates consistent with pulmonary edema. Electrocardiogram was notable for anterolateral ST depressions and ST elevations in aVR and V1 concerning for left main disease. Bedside echocardiography demonstrated depressed left ventricular function with an estimated ejection fraction of 30% and wall motion abnormalities in the left anterior descending coronary artery distribution. Her APACHE II score at that time was 36, corresponding to a predicted mortality rate of 82%. Given the conflicting need for high-dose pressors and evidence of myocardial ischemia potentially made worse by the same pressors, the decision was made to pursue coronary angiography and consideration for mechanical hemodynamic support. Left heart catheterization revealed proximal left main, severe ostial left anterior descending artery disease of 70%, and moderate right coronary artery disease (Figure 1). Right heart catheterization revealed elevated mean pulmonary artery pressure of 32 mmHg, wedge pressure of 18 mmHg, and low-normal cardiac index of 2.95 L/min per square meter (L/min/m^2^) while on high-level vasopressors. Although no acute plaque rupture was noted on angiography, her ostial left anterior descending artery disease was thought to be the likely site responsible for the myocardial ischemia contributing to her shock. Because percutaneous coronary intervention (PCI) of this high-risk lesion in a critically ill patient could lead to further instability, PCI was deferred and mechanical circulatory support was initiated with an Impella 2.5 device (Abiomed, Inc., Danvers, MA, USA). Postintervention laboratory indices and urine output improved within minutes along with decreasing vasopressor requirements. The Impella delivered an average 2 L/min of flow and was removed at approximately 72 hours, after vasopressors were weaned off entirely (Figure 2).

After a total of one week in the ICU for invasive hemodynamic and respiratory support, the patient was transferred to the medical floor on supplemental oxygen by nasal cannula. During that time, her lactate, troponin, and creatinine normalized. Final blood and sputum cultures were negative, but urine cultures identified an* Escherichia coli* urinary tract infection as the likely source of her sepsis. The patient underwent 2-vessel coronary artery bypass graft two weeks from her initial presentation and was discharged to a rehabilitation facility. Her outpatient anemia evaluation identified a colonic adenocarcinoma, for which she received a successful right hemicolectomy three months after discharge. A phone call over one year following discharge found her to be in good spirits and functioning independently at home.

## 3. Discussion

In this case, we describe the use of an Impella 2.5 device to provide mechanical circulatory support to a patient with coronary artery disease and anemia presenting with distributive septic shock from a urinary tract infection, further complicated by ischemic cardiogenic shock. The patient's hemodynamic compromise despite maximal medical therapy and active myocardial ischemia, as evidenced by electrocardiogram and marked troponin elevation, led to an invasive cardiac evaluation. Severe left main disease on left heart catheterization provided supportive evidence for the contribution of ongoing myocardial ischemia to her septic shock. The acuity and degree of her illness warranted immediate mechanical circulatory support in an attempt to halt the cycle of cardiogenic shock and ongoing ischemia refractory to inotropic and vasopressor therapy. Device deployment led to rapid improvement of serum lactate, decreased vasopressor requirements, and resumption of urine output within 15 minutes. This rate of recovery of renal function is biologically plausible and analogous to the timing of onset of urine production observed during renal transplantation [5]. Mechanical augmentation of cardiac output and aggressive diuresis also led to brisk improvement of respiratory function, as evidenced by decreasing ventilator requirements and prompt clearance of bilateral opacities on serial chest radiography. In this context, the source of respiratory distress was more consistent with cardiogenic pulmonary edema than from a primary infectious respiratory source.

Although sepsis is not an absolute contraindication to mechanical hemodynamic support, using a foreign device in a septic patient is generally avoided when possible. However, in the described case, the patient's dire need for unloading the myocardium while providing supplemental cardiac output outweighed the concern for introducing a new nidus of infection. The successful utilization of the Impella and favorable outcome in this patient suggest a possible role for such mechanical hemodynamic support devices in patients with cardiac ischemia complicating sepsis. Currently, therapeutic options for critically ill patients with refractory shock are limited to cardioactive medications (i.e., vasopressors, inotropes), intra-aortic balloon pump (IABP), extracorporeal life support, and now the utilization of microaxial pumps such as the Impella.

While primarily used for cardiogenic shock and support during percutaneous coronary intervention, the use of the Impella has also recently been described in transcatheter aortic valve replacement [6] and fulminant myocarditis [7, 8]. Its two smaller percutaneous models, the Impella 2.5 and Impella CP, provide 2.5 and 4.0 L/min of support, respectively, and its larger surgically implanted model, the Impella 5.0, delivers 5 L/min of support. The Impella CP is frequently favored due its smaller size and relative ease of insertion in the common femoral artery with a 14-French introducer sheath via Seldinger technique under fluoroscopic guidance in the catheterization laboratory. In contrast, the Impella 5.0 requires surgical cut-down to the axillary artery and a cardiac surgery team for device insertion. Its use is contraindicated in the presence of a prosthetic aortic valve, aortic valve stenosis, moderate-to-severe aortic insufficiency, severe aortic or peripheral artery disease, or severe bleeding diatheses. Despite the potential for complications such as bleeding, hemolysis, and catheter drift, the Impella exhibits safety comparable to the IABP [1, 9, 10].

The Impella has increasingly gained favor due to its ability to augment cardiac output to a greater extent than the IABP [9, 10]. The majority of the literature to date has compared the use of microaxial pump devices, such as the Impella, to IABP in those with primary cardiogenic shock. Although several studies have demonstrated advantages of the Impella, including increased hemodynamic support without significant increases in insertion or postprocedural complications, improved mortality with its use has yet to be established [2, 3, 9]. Despite being halted prematurely for futility based on an interim analysis, the PROTECT II trial, which compared the Impella to IABP in high-risk patients receiving percutaneous coronary intervention, revealed trends toward improved 90-day mortality in the Impella group [2]. Post hoc analysis indicated that the first patients to receive the Impella at each participating site experienced more major adverse effects than those receiving the IABP. However, after excluding the first patient per group at each site, significantly lower 90-day major adverse events were observed with the Impella (38.0% versus 50.0%  [*P* = .029]). This suggests a learning curve associated with its insertion and potential for superior outcomes when utilized by practitioners familiar with the device [11].

A paradigm shift has occurred since the introduction of the microaxial devices. Although less is understood about their medical complications compared to other forms of mechanical support such as surgically implanted ventricular assist devices [12], the relative ease of micro-axial device deployment makes it an appealing option in the proper circumstance. Their use has notably increased in high-risk percutaneous intervention, advanced heart failure, and acute myocardial infarction with or without cardiogenic shock, but use in other clinical scenarios has been fairly limited until recently [13]. While Impella use is well described in postcardiotomy, fulminant myocarditis, and cardiac arrest, we are not aware of prior reports describing Impella use for cardiogenic shock in patients with type 2 NSTEMI secondary to sepsis.

Complex patients like the one presented are not uncommon in the intensive care unit. Epidemiological reports establish patients over the age of 65 now account for 45% of the intensive care unit population [14]. These patients often have numerous comorbidities such as advanced coronary artery disease and anemia predisposing to multifactorial hemodynamic compromise. A recent CDC report estimates the prevalence of coronary artery disease in elderly Americans is almost 20% [15]. Additionally, sepsis disproportionally afflicts the elderly, with nearly 40% of those admitted for septicemia being between the ages of 65 and 84 [16].

Despite advances in intensive care, sepsis remains a leading cause of death in noncardiac intensive care units, with an estimated mortality of 25 to 30% [17, 18]. Shock, whether of septic or cardiac origin, portends a poor prognosis when presenting in isolation and is conceivably worse when diagnosed in tandem. The burgeoning elderly population, which is disproportionately affected by both coronary artery disease and sepsis, will test our therapeutic options and require novel approaches for providing the necessary hemodynamic augmentation to overcome acute shock. For example, although our patient was provided sufficient red blood cells to correct her nonhemorrhagic anemia and appropriate antibiotics for her urosepsis, the degree of her shock was beyond the capability of medical therapy to yield a good outcome. For such patients in whom optimal medical therapy fails for the treatment of multifactorial shock, the Impella may be a viable alternative, even in the setting of sepsis. As with any time-dependent disease process, its early initiation in the judiciously screened patient may yield encouraging results.
